# Supplementary material for: From sequence to enzyme mechanism using multi-label machine learning
Source: BMC Bioinformatics. 2014 May 19;15:150. doi: 10.1186/1471-2105-15-150 (PMC4229970; doi:10.1186/1471-2105-15-150)
Supplement: Additional file 2 — Java code of ml2db. Additional file ml2db_code.tar.gz contains the Java source code to run the multi-label machine learning experiments and save the results to database. The code’s Javadoc is included. [file 1471-2105-15-150-S2.zip › additional file 2/ml2db/ml2db/doc/mulan/evaluation/measure/MicroPrecision.html]

MicroPrecision


JavaScript is disabled on your browser.


- Overview
- Package
- Class
- Use
- Tree
- Deprecated
- Index
- Help

- Prev Class
- Next Class

- Frames
- No Frames

- All Classes

- Summary:
- Nested |
- Field |
- Constr |
- Method

- Detail:
- Field |
- Constr |
- Method


mulan.evaluation.measure

## Class MicroPrecision

- java.lang.Object
- - mulan.evaluation.measure.MeasureBase
  - - mulan.evaluation.measure.BipartitionMeasureBase
    - - mulan.evaluation.measure.LabelBasedBipartitionMeasureBase
      - - mulan.evaluation.measure.LabelBasedPrecision
        - - mulan.evaluation.measure.MicroPrecision

- All Implemented Interfaces:
  :   java.io.Serializable, Measure

  ---

    

  ```
  public class MicroPrecision
  extends LabelBasedPrecision
  ```

  Implementation of the micro-averaged precision measure.

  Version:
  :   2010.11.05

  Author:
  :   Grigorios Tsoumakas

  See Also:
  :   Serialized Form

- - ### Constructor Summary

    Constructors

    Constructor and Description

    `MicroPrecision(int numOfLabels)`

    Constructs a new object with given number of labels
  - ### Method Summary

    Methods

    | Modifier and Type | Method and Description |
    | --- | --- |
    | `java.lang.String` | `getName()` Gets the name of a measure. |
    | `double` | `getValue()` Gets the value of a measure. |

    - ### Methods inherited from class mulan.evaluation.measure.LabelBasedPrecision

      `getIdealValue`
    - ### Methods inherited from class mulan.evaluation.measure.LabelBasedBipartitionMeasureBase

      `reset, updateBipartition`
    - ### Methods inherited from class mulan.evaluation.measure.MeasureBase

      `makeCopy, toString, update`
    - ### Methods inherited from class java.lang.Object

      `equals, getClass, hashCode, notify, notifyAll, wait, wait, wait`

- - ### Constructor Detail


    - #### MicroPrecision

      ```
      public MicroPrecision(int numOfLabels)
      ```

      Constructs a new object with given number of labels

      Parameters:
      :   `numOfLabels` - the number of labels
  - ### Method Detail


    - #### getValue

      ```
      public double getValue()
      ```

      **Description copied from interface: `Measure`**

      Gets the value of a measure. The measure is incrementally cumulated for learner's
      prediction by each `Measure.update(MultiLabelOutput, boolean[])` call. The value
      returned by the method, returns sum of all update calls divided by the number
      of calls (average of all measures for all predictions).

      Returns:
      :   the average measure value computed so far


    - #### getName

      ```
      public java.lang.String getName()
      ```

      **Description copied from interface: `Measure`**

      Gets the name of a measure.

      Returns:
      :   the name of a measure.


- Overview
- Package
- Class
- Use
- Tree
- Deprecated
- Index
- Help

- Prev Class
- Next Class

- Frames
- No Frames

- All Classes

- Summary:
- Nested |
- Field |
- Constr |
- Method

- Detail:
- Field |
- Constr |
- Method
